# L’inflation du ballonnet de la sonde d’intubation par la lidocaïne alcalinisée réduit les douleurs laryngées post-opératoires: essai clinique prospectif contrôlé randomisé

**DOI:** 10.11604/pamj.2022.41.230.31156

**Published:** 2022-03-21

**Authors:** Dounies Choura, Salma Ketata, Imen Zouche, Amine Zouari, Hassen Moalla, Yassine Maktouf, Ameni Marwene, Zied Triki

**Affiliations:** 1 Service d'Anesthésie-Réanimation Chirurgicale, Centre Hospitalier Universitaire Habib Bourguiba Sfax, Sfax, Tunisie,; 2 Service de Chirurgie Viscérale, Centre Hospitalier Universitaire Habib Bourguiba Sfax, Sfax, Tunisie,; 3 Service de Stomatologie et de Médecine Dentaire, Hôpital Régional de Mahres Sfax, Sfax, Tunisie

**Keywords:** Anesthésie générale, intubation orotracheal, lidocaïne, General anesthesia, orotracheal intubation, lidocaine, sore throat after intubation

## Abstract

**Introduction:**

les douleurs laryngées post-opératoires (DLPO) sont fréquentes et gênantes. Le but de notre travail était d´évaluer l´efficacité de l´inflation de la lidocaïne alcalinisée dans le ballonnet de la sonde d´intubation orotrachéale (SIOT) dans la prévention des DLPO.

**Méthodes:**

nous avons mené une étude prospective monocentrique randomisée en double aveugle incluant des patients subissant une anesthésie générale avec IOT de durée prévue inférieure à 240 min et répartis en deux groupes équivalents: groupe lidocaïne (GL): patients dont le ballonnet de la SIOT est gonflé par la lidocaïne alcalinisée et groupe Témoin (GT): patients dont le ballonnet de la SIOT est gonflé par du sérum physiologique. Le critère de jugement principal était l´incidence des DLPO au premières 24 heures postopératoires et les critères de jugement secondaires étaient l´incidence de la toux, la dysphonie et des nausées vomissement post opératoires NVPO durant les premières 24 heures post opératoires. L´analyse statistique était réalisée par le logiciel SPSS version 25. La différence était considérée significative lorsque p < 0,05.

**Résultats:**

nous avons inclus 60 patients randomisés en 2 groupes de 30. Les 2 groupes étaient comparables par rapport à leurs critères démographiques et anesthésiques. L´inflation du ballonnet de la SIOT par la lidocaïne alcalinisée réduit l´incidence des DLPO, de la toux, de la dysphonie et des NVPO. Aucun cas de rupture du ballonnet ou de toxicité à la lidocaïne n´a été rapporté.

**Conclusion:**

l´inflation du ballonnet de la SIOT par la lidocaïne alcalinisée prévient les DLPO.

## Abstract (en)

**Introduction:**

post-operative laryngeal pain (POLP) is frequent and embarrassing. The purpose of this study is to assess the efficacy of endotracheal tube balloon (ETB) inflation with alkalinized lidocaine in the prevention of the POLP.

**Methods:**

we conducted a prospective, single-center, double-blind, randomized study including patients undergoing general anesthesia with tracheal intubation (OTI) with an expected duration of less than 240 min. Patients were divided into two equivalent groups: lidocaine (GL) group: patients with ETB inflated with alkaline lidocaine and the control group (GT): patients whose ETB was inflated with normal saline solution. The primary endpoint was the incidence of POLP in the first 24 hours after surgery and the secondary endpoint was the incidence of cough, dysphonia and postoperative vomiting, postoperative nausea and vomiting (PONV) in the first 24 hours after surgery. Statistical analysis was carried out using SPSS software version 25. The difference was considered significant when p< 0.05.

**Results:**

the study included 60 patients randomized into 2 groups of 30 patients each. Both groups were comparable with regard to their demographic and anesthetic criteria. ETB inflation with alkalinized lidocaine reduced the incidence of POLP, cough, dysphonia and PONV. No cases of balloon rupture or lidocaine toxicity were reported.

**Conclusion:**

ETB inflation with alkaline lidocaine prevents POLP.

## Introduction

Les complications laryngées, après anesthésie générale (AG) notamment la dysphonie, la dyspnée laryngée et les douleurs laryngées sont gênantes et de fréquence variable [1, 2]. Les douleurs laryngées sont rapportées dans 50% des cas et peuvent être responsable de bronchospasme, de pic hypertensif, de tachycardie, d´arythmies, de sensation désagréable et même de mouvements dangereux pour le malade [3, 4]. Afin de réduire l´incidence des effets indésirables liés à l´intubation orotrachéale (IOT) plusieurs stratégies ont été proposées comme le recours aux antalgiques et aux anti-hyperalgésiques systémiques [5], la corticothérapie systémique ou l´instillation locale des gels et sprays de corticoïdes [5, 6], le gargarisme par la kétamine ou les anti-inflammatoires [7], ainsi que le contrôle de la pression du ballonnet [1, 8]. Toutefois, ces méthodes restent limitées avec des résultats parfois insuffisants. L´objectif de notre étude était d´évaluer l´intérêt de la lidocaïne alcalinisée, comme solution de gonflement du ballonnet dans la prévention des douleurs laryngées post-opératoire.

## Méthodes

**Conception de l´étude:** après avoir obtenu l´accord du comité d´éthique local et le consentement éclairé et écrit des patients, nous avons mené une étude prospective contrôlée randomisée en double aveugle débutée le 01 Mars 2019 au service d´anesthésie réanimation du CHU Habib Bourguiba Sfax portant sur des patients subissant une anesthésie générale avec IOT.

**Critères d´éligibilité:** nous avons inclus les patients dont l´âge était supérieur à 18 ans classés ASA 1, 2 ou 3 et proposés pour chirurgie abdominale, urologique, réparatrice (non orale ou cervicale) ou orthopédique programmée avec IOT prévue facile et dont la durée était inférieure à 240 minutes. Les patients avec des difficultés à l´intubation, les patients extubés accidentellement ou réintubés, les patients intubés de façon traumatique ou ayant présentés une réaction allergique et avec qui on a eu recours aux glucocorticoïdes ou des antiémétiques ont été exclus.

### Randomisation

A l´entrée au bloc opératoire, la randomisation a été faite par un 1^er^ médecin anesthésiste qui n´a pas intervenu dans le déroulement de l´étude suivant une séquence générée par le site: www.sealedenvelope.com. Les patients ont été répartis en deux groupes équivalents: Groupe Lidocaïne (GL): patients dont le ballonnet de la SIOT était gonflé par un volume ne permettant aucune fuite d´air fait de solution de 40 mg de lidocaïne 2% alcalinisée par 4,2 mg de bicarbonate (concentration de 4mg/ml de lidocaïne). Groupe Témoin (GT): patients dont le ballonnet de la SIOT était gonflé par un volume ne permettant aucune fuite d´air fait de sérum physiologique.

### Déroulement de l´étude

A la salle opératoire, tous les patients étaient monitorés par un électrocardioscope, une pression artérielle non invasive et un saturomètre. Une voie veineuse 20 gauge a été mise en place et un pré remplissage par 10cc/kg de sérum physiologique a été débuté. Le protocole d´anesthésie a été standardisé pour tous les patients avec une induction au fentanyl 3 µg.kg-1et propofol 3 mg kg-1 et de 0,15 mg.kg-1 de Cisatracurium. L´intubation orotrachéale a été faite par un 2^e^ médecin anesthésiste expérimenté à l´aide d´un laryngoscope réutilisable et par une sonde d´intubation orotrachéale SIOT de la marque « BICAKCILAR® » avec ballonnet semi-perméable de volume standard, à faible pression. On a utilisé des SIOT de 7 mm de diamètre interne pour les femmes (Référence 551 0070 1) et de 7,5 mm de diamètre interne pour les hommes (Référence 551 0075 1). L´inflation du ballonnet de la sonde a été faite par un 3^e^ médecin anesthésiste, qui ne connait pas la nature de la solution, d´une façon lente par le liquide préconisée jusqu´à ne plus écouter le bruit d´une fuite d´air permettant ainsi une pression intraballonnet estimée aux alentours de 20mmHg. L´entretien anesthésique a été assuré par du sevoflurane 2% et des réinjections de 1µg/kg de fentanyl et 0,05mg /kg de cisatracrium toutes les 30 minutes.

Tous les patients étaient ventilés en mode ventilation assistée contrôlée (VAC) avec un volume courant (VT) de 6 ml/kg de poids idéal théorique (PIT) pour une Pet CO_2_ entre 35-40mmhg. La FiO_2_ était fixée à 50% avec un mélange O_2_/AIR et la pression télé-expiratoire positive était fixée entre 4 et 6mmHg. L´analgésie post-opératoire a été initiée 30 minutes avant la fin de l´acte par 1g de paracétamol, 20 mg de néfopam et 100mg de tramadol. L´extubation a été réalisée sur table opératoire chez un patient bien éveillée bien analgésié réchauffé et décurarisé. Les patients ont été transférés en SSPI pour surveillance post-opératoire pendant 2 heures puis aux services d´hospitalisation correspondants. La surveillance post-opératoire était assurée par un autre anesthésiste qui ne connaît pas les groupes randomisés. L´analgésie post-opératoire par voie systémique a été assurée aux services d´hospitalisation par du paracétamol 1g x 4, néfopam 20mg x 4 et tramadol 100mg x 3 par jour.

**Le recueil des données:** on a recueilli les données démographiques (âge, sexe, IMC), les ATCD médicaux, le score ASA, le type de la chirurgie, la dose de fentanyl pendant l´acte opératoire, la durée de l´IOT, Echelle visuelle Analogique (EVA) pour les douleurs laryngées à H6 et à H24 post-opératoire et l´incidence de nausée vomissement, de toux et de dysphonie à H6 et à H24. La survenue de rupture du ballonnet ou de signe de toxicité à la lidocaïne ont été recherchés.

**Les critères de jugement:** le critère de jugement principal était l'incidence de la douleur laryngée à la sixième heure post-opératoire. Les critères de jugements secondaires étaient: l´incidence de la douleur laryngée à la vingt-quatrième heure en post-opératoire, l´incidence de toux, de dysphonie et des nausées et vomissements post-opératoires (NVPO) à H6 et H24.

### Analyse statistique

Le calcul de la taille de l'échantillon a été effectué sur la base d'une étude pilote précédente par l´équipe de Hirota *et al*. [9]. Nous avons estimé que l'utilisation de lidocaïne réduirait le taux des maux de gorge de 50% à H6 par rapport au groupe témoin. Sur la base de ces estimations, nous avons calculé une taille d'échantillon permettant une erreur de type I α = 0,05 avec une puissance de 80%. Il a été estimé nécessaire que chaque groupe soit formé par un minimum de 25 individus. Nous avons choisi d´inclure 60 patients répartis sur deux groupes de 30 individus. L´analyse statistique a été faite à l´aide du logiciel SPSS version 25. Les variables qualitatives ont été exprimées en pourcentage. Les variables quantitatives ont été exprimées en moyenne ± écart type après avoir vérifié la normalité de la distribution par le test shapiro-Wilk. Dans le cas contraire (distribution non gaussienne) les valeurs ont été rapportées en médiane avec la plage interquartile (25% et 75%). La comparaison des variables quantitatives a été réalisée par le test t student lorsqu´elles suivent une distribution normale ou par le test Mann Whitney lorsqu´elles suivent une distribution non normale. La comparaison des variables qualitatives a été réalisée par le test de chi deux. La différence était considérée comme significative lorsque p < 0,05.

**Considération éthique:** notre essai clinique a été réalisé après accord préalable du comité de protection des personnes du sud (CPPSUD) sous l´égide des ministères de la santé et de la justice de la république tunisienne et après consentement éclairé et écrit des patients inclus.

## Résultats

**Figure 1:**
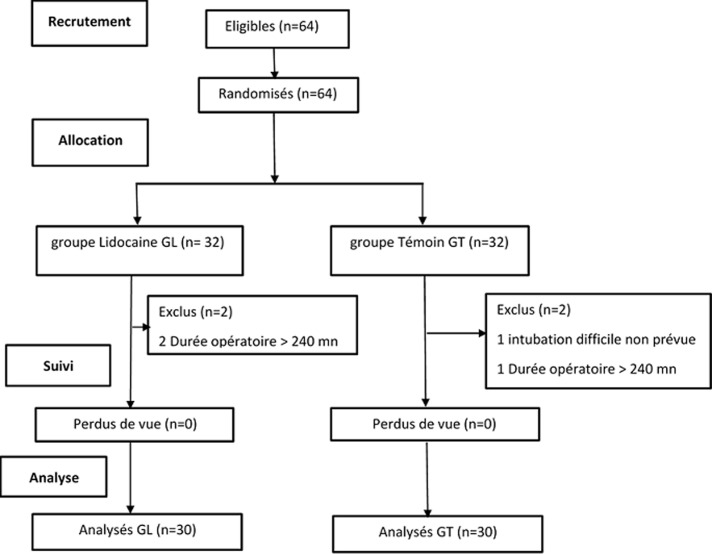
diagramme de flux

L´étude a duré quatre mois du 1^er^ mars au 1^er^ juillet 2019. Nous avons inclus 64 patients randomisés en 2 groupes (groupe GL et groupe GT), 4 patients ont été exclus (1 pour intubation difficile imprévisible et 3 pour une durée opératoire dépassant les 240 minutes) et 60 ont été analysés répartis en 30 patients groupes (Figure 1).

**Caractéristiques générales:** l´âge moyen dans notre population était 68±16,59 ans avec un sex-ratio H/F égal à 0,66. Les deux groupes étaient comparables par rapport à leurs critères démographiques, leurs antécédents, le type de chirurgie, la durée de la chirurgie, et la dose totale fentanyl (Tableau 1). Il n'y avait pas de différence ni dans le volume initial injecté dans le ballonnet à l´intubation ni dans le volume retiré du ballonnet à l´extubation.

**Tableau 1:** comparaison des paramètres démographiques et anesthésiques entre les 2 groupes

	Groupe GL (N=30)	Groupe GT (N=30)	P
**Sexe (H/F)**	13/17	11/19	0.20 ƚ
**Age (ans)±ET**	49,83±17,329	47,03±16,372	0.53*
**Poids (Kg)**	74 ,83[50-120]	75,83[60-120]	0.89‡
**Taille (cm)**	166,7[148-180]	167,7[150-187]	0.90‡
**Score ASA**			
**ASA I**	18	16	0.80 ƚ
**ASA II**	9	11	
**ASA III**	3	3	
**HTA**	7	5	0.74 ƚ
**Diabète**	5	5	0.99 ƚ
**Tabac**	7	7	0.99 ƚ
**BPCO**	5	4	0.71 ƚ
**Durée moyenne de l´intervention (min) ±ET**	123±26,42	127±25,3	0.82*
**Durée moyenne de l´IOT (min) ±ET**	115±24,56	116±25,17	0,53*
**Dose moyenne de fentanyl (µg/kg) ±ET**	4,28±0,804	3,97±0 ,743	0,123*

H: Homme, F: femme, N= nombre; ET: Ecart type, [ ]: extrêmes, ƚ: test de chi deux, *: test de t student, ‡: test U de Mann Whitney, GL : groupe lidocaïne, GT: groupe Témoin, HTA: hypertention artérielle, BPCO: bronchopneumopathie obstructive, IOT: intubation orotrachéale, cm: centimètre, µg: microgramme, kg: kilogramme, min: minute

**Incidence des douleurs laryngées post-opératoires:** dans le groupe GL il y avait une diminution statistiquement significative de l´incidence des douleurs laryngées à la sixième heure (p = 0,004) et à la vingt-quatrième heure post-opératoire (p = 001) par rapport au groupe GT (Tableau 2).

**Tableau 2:** comparaison de la douleur laryngée à H6 et à H24 entre les 2 groupes

		Groupe GL	Groupe GT	P
**DOULEURS H 06**	EVA>3	9	20	0,004ƚ
	EVA≤3	21	10	
**DOULEURS H 24**	EVA>3	4	20	0,001ƚ
	EVA≤3	26	10	

GL: groupe Lidocaïne, GT: groupe Témoin, EVA: Echelle Visuelle Analogique, ƚ: test de chi deux

### Incidence de la toux, la dysphonie et des NVPO

Il n´y avait pas de différence significative entre les 2 groupe concernant l´incidence de la toux à l´intubation avec p 0,136 cependant la différence devenait significative à l´émergence de l´anesthésie, à H6 et H24 post-opératoire avec des p respectivement =0,001, 0,04 et 0,001 (Tableau 3). L´incidence de la dysphonie à H06 et à H24 a diminué d´une façon significative dans le groupe GL par rapport au groupe GT (p = 0,001, p < 0,001 respectivement). Quant aux NVPO, il n´y avait pas de différence significative entre les 2 groupes à H6 (p=0,149) par contre il y avait une diminution statistiquement significative dans le groupe GL à H 24avec p = 0,018 (Tableau 3). Aucun cas de rupture du ballonnet n´a été colligé et aucun signe de toxicité à la lidocaïne n´a été rapporté.

**Tableau 3:** comparaison de l´incidence de la toux, la dysphonie et des NVPO entre les 2 groupes

	Groupe GL	Groupe GT	P value
**Toux à l´intubation**			
**Toux +/-**	5/25	10/20	0.136 ƚ
**Toux à l´émergence de l´anesthésie**			
**Toux +/-**	5/25	22/8	<0.001 ƚ
**Toux à H6**			
**Toux +/-**	8/22	19/11	0.004 ƚ
**Toux à H24**			
**Toux +/-**	4/26	16/14	0.001 ƚ
**Dysphonie H6**			
**Dysphonie +/-**	11/19	26/4	0.001 ƚ
**Dysphonie H24**			
**Dysphonie +/-**	7 /23	26/4	<0.001 ƚ
**NVPO H6**			
**NVPO +/-**	14/16	19/11	0.149 ƚ
**NVPO H24**			
**NVPO +/-**	8/22	17/13	0.018 ƚ

GL: groupe Lidocaïne, GT :groupe Témoin ,+/-: présent / absent, ƚ: test de chi deux , NVPO: nausée vomissement post-opératoire

## Discussion

Nous avons menée une étude prospective randomisée en double aveugle portant sur des patients proposés pour des chirurgies sous anesthésie générale avec IOT de durée prévue inférieure à 240 minutes dans le but d´évaluer l´efficacité de l´inflation de la lidocaïne alcalinisée dans le ballonnet de la sonde d´intubation orotrachéale SIOT dans la prévention des Douleurs laryngées post-opératoires. Notre étude a montré que l´inflation du ballonnet de la SIOT à l´aide d´une solution 4mg/ml de lidocaïne alcalinisée pourrait réduire notamment l´incidence des douleurs laryngées post intubation et limiter l´incidence de la toux, la dysphonie et des NVPO. Nous avons également montré que cette stratégie était dénuée des risques de rupture du ballonnet ou de toxicité systémique à la lidocaïne. les propriétés antalgiques et antitussives de la lidocaïne administrée en IV ont été prouvées sans pour autant être associée à des effets indésirables notables [9, 10] et ceci pendant l´IOT, l´extubation, la bronchoscopie et la bronchographie [11-13]. L´application locale de la lidocaïne a la particularité d´exercer son effet antalgique et antitussif par deux mécanismes; premièrement, elle peut être absorbée par la muqueuse des voies aériennes pour atteindre des concentrations adéquates au niveau du plasma et agir d´une façon systémique et secondairement, elle peut exercer ses propriétés d´anesthésique local en bloquant la transmission nerveuse des proprio-récepteurs d´adaptation lente situés au niveau du larynx et de la trachée, dont la stimulation autant au moment de l´intubation que de l´extubation, est à l´origine des réflexes protecteurs des voies aériennes supérieures (VAS) dont notamment la toux [14].

La revue de la littérature nous a montré que le seuil de concentration plasmatique de lidocaïne pour atténuer les réflexes des VAS est de 3 µg/ml quand elle est utilisée par voie IV [15], cette molécule est efficace pour bloquer ces réflexes quand elle est utilisée en spray intra-trachéal avec une concentration plasmatique mesurée inférieure à ce seuil [16]. De ce fait, l´effet local de la lidocaïne parait être le principal mécanisme impliqué. Nos résultats, en ce qui concerne la diminution de la douleur laryngée, sont en accord avec une méta-analyse publiée par Tanaka *et al*. [17] évaluant l´intérêt de la lidocaïne, avec ses différentes voies d´application, pour la prévention des maux de gorge en postopératoire. Cette méta-analyse a porté sur15 études dont 7 avaient utilisé la lidocaïne pour l´inflation du ballonnet, soit un effectif de 381 patients. La sévérité des douleurs pharyngées était réduite avec une différence moyenne qui tend vers la significativité statistique. Lam *et al*. [18] ont inclu 1566 patients à travers 19 études et s´intéressant plus spécifiquement à l´inflation du ballonnet de la SIOT par la lidocaïne a évalué l´intérêt de cette stratégie sur les phénomènes d´émergence. Onze études, soit un effectif de 744 patients, ont porté sur les douleurs pharyngolaryngées post-opératoires dont 9 études ayant eu recours à la lidocaïne alcalinisée. Parmi ces 9 études, 8 comportaient un groupe contrôle utilisant le sérum physiologique. Les résultats ont montré que la lidocaïne alcalinisée offrait une protection contre les douleurs pharyngées post-opératoires avec un RR=0,33 [IC 95%: 0,22 à 0,5].

Dans la première application clinique de cette stratégie, Dollo et Estebe [19] ont pu montrer chez un petit effectif de 15 patients que l´alcalinisation de petites quantités de lidocaïne (20, 30 et 40 mg) était efficace pour assurer une meilleure tolérance de la SIOT (évaluée par l´allongement du temps de ventilation spontanée avant l´extubation). Depuis les résultats de cette étude pilote, la même équipe a confirmé, chez 60 patients proposés pour chirurgie lombaire, l´efficacité de l´alcalinisation d´une dose de 40 mg de lidocaïne pour diminuer l´incidence de la toux à l´émergence de l´anesthésie) [20].

De même, Souissi *et al*. [21] ont montré dans une étude regroupant 80 patientes proposées pour chirurgie gynécologique que l´utilisation de la lidocaïne alcalinisée pour l´inflation du ballonnet réduit d´une façon significative la survenue de la toux comparativement à un groupe où l´inflation a été faite à l´aide de sérum physiologique. Parmi les limites de notre étude, nous n´avons pas pu monitorer objectivement la pression du ballonnet de la SIOT vue l´absence d´un manomètre à eau dans notre centre et la quasi-totalité des manomètres dans le marché sont des manomètres permettant de mesurer la pression des ballonnets remplis d´air. Par ailleurs, notre étude est monocentrique avec une taille d´échantillon limitée et puissance faible 80%. Une étude plus large aurait eu plus de sensibilité à détecter un impact surtout vis-à-vis des critères de jugement secondaires.

## Conclusion

La lidocaïne alcalinisée dans le ballonnet est une méthode efficace pour diminuer la morbidité laryngée post-opératoire liée à l´intubation orotrachéale et pourrait être une alternative chez les patients de réanimation.

### Etat des connaissances sur le sujet

- Les maux de gorge sont devenus des plaintes communes et fréquentes après l´anesthésie générale et qui altèrent la satisfaction des patients après la chirurgie ainsi contrôler ces morbidités est devenue obligatoire pour avoir une procédure anesthésique de qualité;
- La lidocaïne est un anesthésique local et antihyperalgésique très utile pour lutter contre la douleur.

### Contribution de notre étude à la connaissance

- La lidocaïne alcalinisée dans le ballonnet permet une faible irritation des voies aérienne et donc une diminution des douleurs laryngés de la toux et de la dysphonie post opératoire;
- La lidocaïne alcalinisée dans le ballonnet peut être un moyen efficace pour lutter contre les nausées, vomissement post opératoires.
